# Pharmacoeconomic evaluation of voriconazole vs. liposomal amphotericin B in empiric treatment of invasive fungal infections in Turkey

**DOI:** 10.1186/1471-2334-13-560

**Published:** 2013-11-26

**Authors:** Stuart J Turner, Esin Senol, Ates Kara, Daoud Al-Badriyeh, Ener C Dinleyici, David CM Kong

**Affiliations:** 1 Department of Pharmacy Practice and Administration, Ernest Mario School of Pharmacy, Rutgers University, New Jersey, USA; 2 Department of Infectious Disease, Gazi University, Ankara, Turkey; 3 Department of Pediatric Infectious Disease, Hacettepe University, Ankara, Turkey; 4 College of Pharmacy, Qatar University, Doha, Qatar; 5 Department of Pediatrics, Pediatric Intensive Care and Pediatric Infectious Disease, Faculty of Medicine, Eskisehir Osmangazi University, Eskisehir 26480, Turkey; 6 Centre for Medicine Use and Safety, Faculty of Pharmacy and Pharmaceutical Sciences, Monash University (Parkville Campus), 381 Royal Parade, Parkville, Victoria 3052, Australia

**Keywords:** Antifungal agents, Voriconazole, Liposomal amphotericin B, Economic evaluation, Invasive fungal infection, Empiric therapy

## Abstract

**Background:**

Invasive fungal infections (IFI) are associated with considerable expense and mortality on healthcare systems. There is a need to provide evidence of both clinical efficacy and value for money with any health technology. The current pharmacoeconomic evaluation investigated the use of liposomal amphotericin B (LAmB) and voriconazole for the empiric treatment of IFI in the Turkish setting.

**Methods:**

Decision analytic modelling was used to create a pathway for patient treatment with a 5-point composite outcome measure. The data was obtained from a major non-inferiority multicentre randomised controlled study, with an expert panel of clinicians in Turkey providing transition probabilities and cost not available in the literature. Sensitivity analyses were performed on the inputs from the clinical trial and the expert panel.

**Results:**

As per the base case analysis, voriconazole was preferred by Turkish Lira (TL) 2,523 per patient treated and TL2,520 per surviving patient. LAmB was the preferred alternative by TL5,362 per successfully treated patient. Removing fever resolution as part of the composite outcome measure resulted in voriconazole being the preferred alternative per successfully treated patient. Univariate sensitivity analysis highlighted that increasing the duration of voriconazole by >1.2 days or decreasing LAmB by >1.0 days changes the result. Monte Carlo Simulation resulted in 69.4% of simulations favouring voriconazole per patient treated.

**Conclusion:**

There is a strong likelihood that voriconazole is economically more favourable than LAmB in the empiric treatment of IFI in Turkey.

## Background

Invasive fungal infections (IFIs) are predominantly an adverse outcome associated with an immunocompromised health state [1]. This is often encountered in prolonged neutropenia resulting from chemotherapy treatments [1]. Indeed, the cost to the healthcare systems for treating IFIs (including antifungal medications, extended hospital stays and other monitoring costs) are of significant concern [2,3].

Liposomal amphotericin B (LAmB) has been a mainstay in the empiric treatment of IFI [4,5]. Other agents include echinocandins such as micafungin, caspofungin and anidulafungin, and azoles such as voriconazole (despite voriconazole having not been approved for empiric use to date) [6-9]. Significantly, all currently used antifungal agents are costly, with the treatment duration being upwards of 1-2 weeks [2].

There are previous economic evaluations of voriconazole and LAmB [10-12]. The majority of these studies highlighted voriconazole as the preferred option. It is important to highlight, however, that the choice of the methodology in these studies may have influenced the conclusion reached. The single study that found LAmB to be economically favourable utilized a model that described treatment beyond the first-line empiric therapy by including alternative scenarios [10]. Other studies have focused on specific antifungal-associated adverse effects, or have used chart review information from a single site as opposed to multi-site data from generalizable sources [11,13]. Of note is that the economic impact of the antifungal agent alone (i.e. drug acquisition cost) does not necessarily reflect the overall relative cost of treatment from a cost-effectiveness perspective, as this requires inclusion of both efficacy data as well as other cost categories [14]. Importantly, economic conclusions are usually not generalizable between countries with different healthcare systems, reimbursement policy and/or standard of care.

The objective of the current study is to determine the cost-effectiveness of voriconazole and LAmB from the Turkish healthcare perspective. The Turkish healthcare system is significantly disparate in functioning and economics to those studied in earlier evaluations. Currently, there are no economic studies which will guide the choice between use of these two agents in the Turkish healthcare or other systems similar to the Turkish settings. As such, an evaluation of voriconazole and LAmB in the empiric treatment of IFI would be useful in providing guidance to clinicians and decision makers working in these settings, with respect to the economic consequences of using these agents.

## Methods

This cost-effectiveness evaluation was constructed using the data from a multicentre, international randomized-controlled non-inferiority study investigating the efficacy and safety of voriconazole vs. LAmB in empiric treatment of IFIs by Walsh et al [3]. The study randomized 415 patients to receive voriconazole and 422 patients to LAmB. Successful treatment was defined using a five-component end point that included: successful treatment of any baseline fungal infection (diagnosed within 24 hours of initiation into the study), absence of a breakthrough fungal infection during therapy (or seven days after completion of therapy), survival for seven days after treatment, no premature discontinuation of therapy, and resolution of fever during the period of neutropenia [3].

### Perspective

The Turkish healthcare system’s perspective was adopted, focusing on direct medical costs from medications related to the empiric treatment of an IFI, hospitalization costs, monitoring and screening tests. Staff wages and costs associated with treating the underlying patient conditions (such as acute myeloid leukaemia) were not included. The time horizon was that used in the Walsh study i.e. up to 7 days beyond the end of therapy [3].

### Model structure

**Figure 1:**
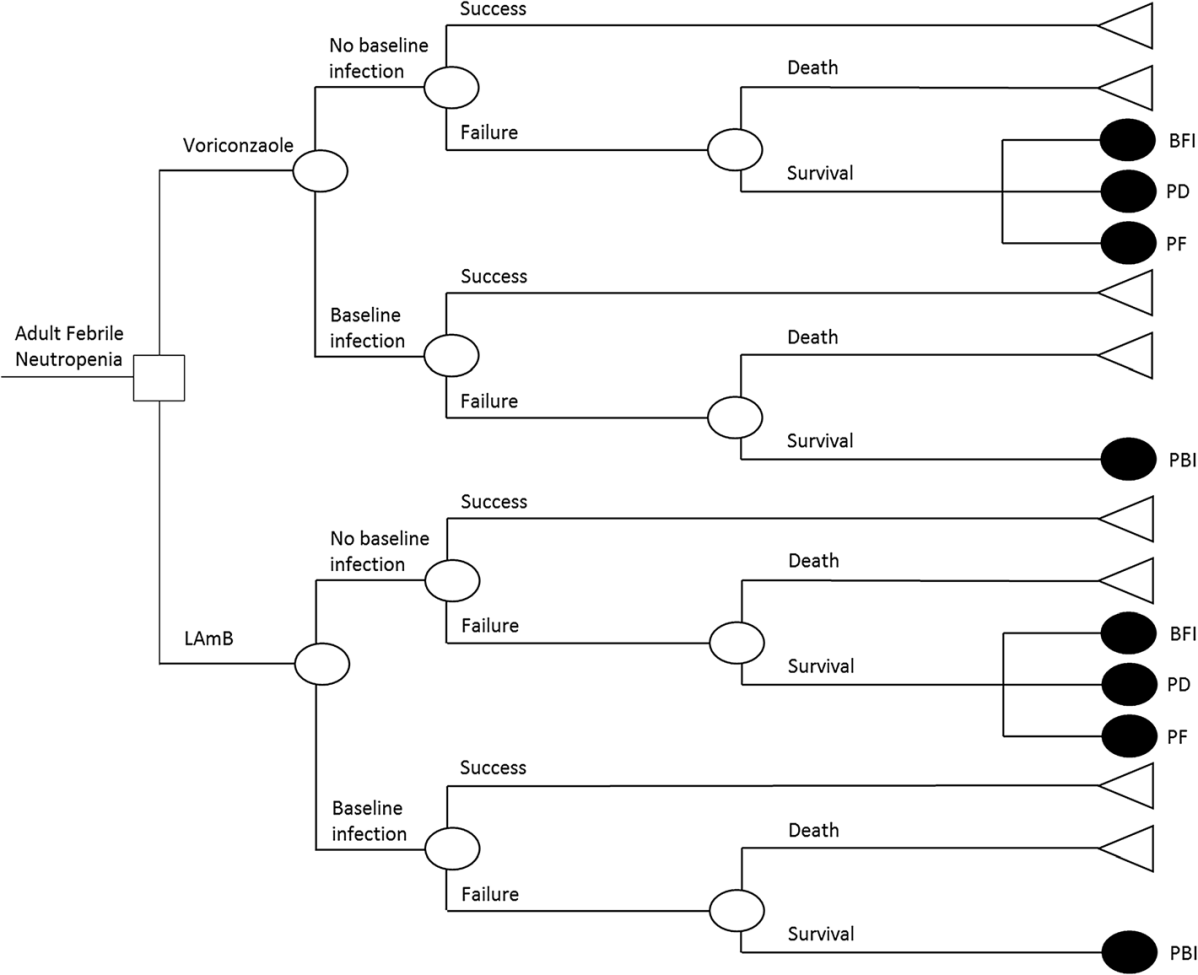
Decision tree for the empiric treatment of IFI with voriconazole or liposomal amphotericin B in adult febrile neutropaenic patients.

The decision-analytic tree outlining the usual treatment algorithm for patients receiving either voriconazole or LAmB was based on work of Al-Badriyeh et al. [10] This tree (Figure 1) allowed for 8 distinct patient outcomes stratified on the presence or absence of a baseline fungal infection; incorporating the 5-point composite outcome measure previously referenced. Those patients with no baseline fungal infection will fall into branches describing either successful treatment from the antifungal agent chosen, or failure resulting in either mortality or distinct outcomes requiring a transition to an alternative agent (breakthrough fungal infection, premature discontinuation or persistent fever). Patients with a baseline fungal infection are described by 3 branches viz. successful treatment, failure resulting in mortality or a persistent baseline fungal infection necessitating a transition to an alternative antifungal agent.

### Model inputs

Clinical data from the Walsh study (Table 1) were used to populate the model including the mortality rate of patients receiving voriconazole and LAmB, the rate of discontinuation due to nephrotoxicity, hepatotoxicity or infusion-related reactions, as well as the occurrence of baseline fungal infections [3]. As per the Walsh study, patients received either 3 mg/kg LAmB intravenously each day or a standard dosing of 6 mg/kg voriconazole every 12 hours for the first two doses then a maintenance dose of 3 mg/kg every 12 hours (or 200 mg orally every 12 hours, after at least 3 days of intravenous therapy). An expert panel of five infectious diseases, haematology and oncology clinicians in Turkey provided a consensus opinion on data required in the model that was not readily available from published or public sources. Information from the panel included duration of antifungal therapy; concomitant medications (related to the antifungal therapy); probability and duration of intensive care unit (ICU) admission, monitoring and screening test utilization; proportion of patients receiving a dose increase or decrease of the initial antifungal agent due to tolerability or lack of efficacy; distribution of discontinuation events; and the alternative antifungal agent given in various scenarios following discontinuation of the initial antifungal agent (Table 2).

**Table 1:** **Clinical outcome data of Voriconazole vs. LAmB for use in decision model **[3]

**Variable**	**Voriconazole**	**LAmB**
Fever with no baseline fungal infection	96.87%	98.58%
Therapeutic success	25.37%	30.05%
Therapeutic failure	74.63%	69.95%
Mortality	11.00%	8.59%
Breakthrough fungal infection	2.67%	7.22%
Premature discontinuation	13.67%	9.62%
Persistent fever^a^	72.67%	74.57%
Fever with baseline fungal infection	3.13%	1.42%
Therapeutic success	46.15%	66.67%
Therapeutic failure	53.85%	33.33%

^a^The number of patients with persistent fever was determined by subtracting the number of patients that failed therapy due to a reason other than persistent fever from the total number of patients that had therapeutic failure to either voriconazole or LAmB.

**Table 2:** Alternative antifungal agents given after treatment failure with Voriconazole or LAmB

**Reason for treatment failure**	**Alternative given**	
	**Voriconazole arm**	**LAmB arm**
Infusion-related reactions^a^	Caspofungin^b^	Voriconazole^c^
Nephrotoxicity	Caspofungin^b^	Caspofungin^b^
Hepatotoxicity	LAmB^d^	Caspofungin^b^
Suspected fungal infection	LAmB^e^	Voriconazole^c^
Persistent fever	LAmB^e^	Voriconazole^c^
**Breakthrough fungal infection**		
*Aspergillus* spp.	LAmB^d^	Voriconazole^c^
*Candida* spp.	Caspofungin^b^	Caspofungin^b^
*Zygomycetes* spp.	LAmB^e^	N/A
DematiaceousMoulds	N/A	LAmB plus posaconazole^f^
Moulds not identified	N/A	LAmB^e^
**Non-responding baseline fungal infection**		
*Aspergillus* spp.	LAmB^d^	Voriconazole^c^
*Candida* spp.	Caspofungin^b^	Caspofungin^b^
*Zygomycetes* spp.	LAmB plus posaconazole^f^	N/A
*Trichoderma fungemia*	N/A	Voriconazole^c^
*Fusarium* spp.	N/A	LAmB plus posaconazole^f^
Moulds not identified	N/A	LAmB plus posaconazole^f^

^a^A switch to alternative therapy due to infusion-related reactions included commencement of pre-dose diphenhydramine; ^b^Caspofungin dose 70 mg on day one, 50 mg thereafter; ^c^Voriconazole dose 6 mg/kg i.v. twice daily on day one, 4 mg/kg i.v. twice daily or 400 mg p.o. twice daily thereafter (22% in base case received oral voriconazole); ^d^LAmB dose 3 mg/kg/day; ^e^LAmB 5 mg/kg/day; ^f^LAmB dose 5 mg/kg/day, posaconazole 200 mg four times per day.

The panel provided consensus opinions that the average treatment duration for both agents (i.e. voriconazole and LamB) was 10 days; piperacillin/tazobactam and vancomycin for the full treatment duration and granulocyte colony-stimulating factor (G-CSF) for the first five days were given as concomitant therapies; 5 days were spent in the ICU out of the total treatment duration by 7.5% of patients; no post-therapy observational period was provided at the end of a course of treatment; dose changes were not seen in the Turkish setting due to adverse effects or lack of efficacy at standard doses, instead a change in antifungal agent was the usual course of action; the common screening tests used during hospitalization included a chest x-ray at onset of fever and weekly thereafter, computed tomography (CT) scan at one week post identification of an unresponsive fever; routine blood cultures and full blood examination (FBE) repeated daily; renal function, liver function and electrolyte tests were performed three times each week; galactomannan assay twice each week and a single bronchoscopy performed in approximately two thirds of all patients. This schedule of screening did not change in the scenario of an ICU admission.

### Assumptions

The assumptions made for our model included:

1. Only one treatment failure may be experienced by a patient which then results in a switch to an alternative antifungal agent or death. The switch to an alternative agent will result in a successful outcome.

2. Due to the inability to characterize the population of patients with baseline fungal infection that had initial treatment failure in the Walsh study [3], all patients in this branch were assumed to survive with persistent unresolved fever.

3. A patient with a baseline fungal infection has the same risk of discontinuation as those with no baseline fungal infections.

4. Mortality and discontinuation events occur at the end of a course of therapy.

### Cost calculations

Costs used were sourced from Sosyal Güvenlik Kurumu (SGK) in Turkey [15]. All costs were reported in 2012 Turkish Lira (TL). To determine the average daily cost of the antifungal medications, an average patient weight of 70 kg was used. This weight was validated by the expert panel. Discounting was not considered due to the short time frame.

Specific costs from the SGK included: LAmB 50 mg vial, TL317; caspofungin 70 mg vial, TL906.99; caspofungin 50 mg vial, TL701.20; voriconazole 200 mg vial, TL284.92; voriconazole 200 mg tablet, TL35.11; a bottle of posaconazole suspension, TL1379.60; G-CSF 300 μg syringe, TL138.50; piperacillin/tazobactam 4.5 g vial, TL23.00; vancomycin 500 mg vial, TL15.73; chest x-ray (per test), TL7.98; diphenhydramine 50 mg vial, TL0.57; CT scan (per test), TL60.50; bronchoalveolarlavage (per lavage), TL177.07; galactomannan assay (per test), TL42.04; routine blood cultures (per test), TL11.00; full blood examination (per test), TL7.18; renal function test (per test), TL10.34; liver function test (per test), TL11.12; electrolyte test (per test), TL4.84; non-ICU per diem, TL79.02; ICU per diem, TL969.52.

For each branch of the decision tree, a weighted average cost was calculated utilizing the total cost and probability associated with each branch. For a patient with successful treatment from the initial antifungal agent, the cost included acquisition of the antifungal agent and concomitant medications, hospitalization, screening and monitoring tests. In the case of failure resulting in death, the cost was equivalent to a successful treatment due to the assumption that any discontinuation and mortality events occur at the end of a course of treatment. For a patient requiring alternative treatment, the total cost includes cost of the alternative antifungal agent and concomitant therapy, hospitalization, screening and monitoring tests, in addition to the cost from the initial therapy.

To reflect clinical practice, all doses of medications were rounded to the nearest full vial. Posaconazole, however, was an exception as it was considered that a bottle of posaconazole suspension could be realistically used for multiple patients. In the situation where costs were provided as a range of options, an average was taken. As patients could be admitted to either a haematological or bone marrow transplant (BMT) wards, a weighted average cost was taken between the two wards, based on the advice of the expert panel as to the proportion of febrile neutropenic patients that would be seen in each ward. Similarly, an average across a 5-day stay in the ICU was taken to reflect the ICU per-diem. The cost of voriconazole therapeutic drug monitoring (TDM) and consequences of voriconazole TDM was not included as the expert panel concluded that voriconazole T DM was rarely performed in the Turkish healthcare system.

### Sensitivity analysis

To determine the model’s robustness, deterministic and probabilistic sensitivity analyses were performed. Univariate sensitivity analyses were conducted on the duration of antifungal treatment (min. 6, max. 14 days), proportion of patients receiving oral voriconazole (min. 0%, max. 50%), proportion of patient utilizing the ICU (min. 5%, max 10%), the daily cost of an ICU bed (min. TL220, max. TL1420) and inclusion of concomitant medications in the overall cost calculations . A threshold analysis based on independent changes on the list price of the initial antifungal agent and duration of treatment was also performed. Univariate sensitivity analysis was conducted to investigate the effect of including/excluding fever resolution as part of the composite outcome measure on the final result, as this had been an area of discussion in recent literature [16,17]. This analysis was achieved by providing an alternate scenario where those patients in branches that would have been considered treatment failures due to persistent fever were instead rolled into the successful treatment branch.

A Monte-Carlo Simulation (MCS) of 10,000 patients was performed to allow variation of several inputs simultaneously, enhancing the study’s generalizability and reflect a situation closer to a real-life situation of multiple simultaneous variations from the base model. The probabilities from the trial by Walsh [3], as well as data provided by the expert panel regarding treatment duration and hospitalization costs were mapped to a distribution of plausible alternatives (Table 3), allowing for a probability of favouring one agent over the other to be developed through iterations of multiple scenarios.

**Table 3:** Input variables and distribution for Monte Carlo simulation sensitivity analyses

**Input variable**	**Voriconazole**	**LAmB**
Fever without baseline infection	Triangular distribution, 92.03%-96.87%-100%	Triangular distribution, 93.65%-98.58%-100%
Therapeutic success	Triangular distribution, 24.10%-25.37%-26.64%	Triangular distribution, 28.55%-30.05%-31.55%
Therapeutic failure	Triangular distribution, 70.90%-74.63%-78.36%	Triangular distribution, 66.45%-69.95%-73.45%
Death	Triangular distribution, 10.45%-11.00%-11.55%	Triangular distribution, 8.16%-8.59%-9.02%
Breakthrough infection	Triangular distribution, 2.40%-2.67%-2.94%	Triangular distribution, 6.50%-7.22%-7.94%
Premature discontinuation	Triangular distribution, 12.30%-13.67%-15.04%	Triangular distribution, 8.66%-9.62%-10.58%
Persistent fever	Triangular distribution, 65.40%-72.67%-79.94%	Triangular distribution, 67.11%-74.57%-82.03%
Fever with baseline infection	Triangular distribution, 2.97%-3.13%-3.29%	Triangular distribution, 1.20%-1.42%-1.49%
Therapeutic success	Triangular distribution, 43.84%-46.15%-48.46%	Triangular distribution, 63.34%-66.67%-70.00%
Therapeutic failure	Triangular distribution, 51.16%-53.85%-56.54%	Triangular distribution, 31.66%-33.33%-35.00%
Ward “per diem”	Discrete, 30% at TL193.40 and 70% at TL30.00	
ICU length of stay	Log-normal, mean 5 days, standard deviation 2.5 days	
Overall treatment duration	Log-normal, mean 10 days, standard deviation 2 days	
Proportion of oral voriconazole use	Triangular distribution, 0-22-50%	

## Results

**Figure 2:**
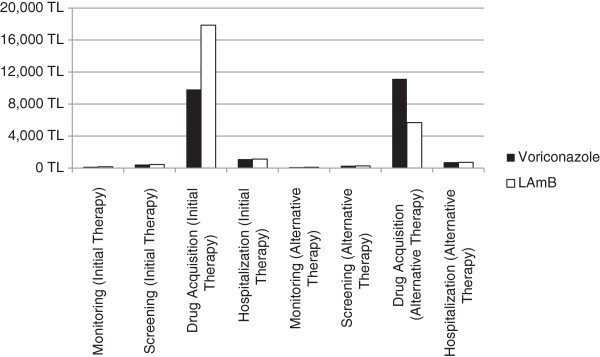
Breakdown by category of cost per patient treated.

Compared to LAmB in the base case analysis, voriconazole was the cost-effective alternative by TL2,523 per patient treated and TL2,520 per surviving patient. LAmB was the preferred alternative by TL5,362 per successfully treated patient. A description of the probability and cost of each branch of the model is given in Table 4. LAmB had a higher probability of success and lower mortality than voriconazole (30.57% vs. 26.02% and 5.92% vs. 7.95%, respectively) [3]. Whilst initial treatment with LAmB is associated with higher cost per patient, the cost associated with alternatives following treatment failure with LAmB is lower compared to using voriconazole (TL17,867 vs. TL9,836 in initial therapy cost and TL5,687 vs. TL11,152 in alternative therapy cost for LAmB vs. voriconazole). The breakdown of the cost categories is described in Figure 2.

**Table 4:** Cost and proportion of patients for empiric use of Voriconazole vs. LAmB

**Outcome**	**Voriconazole**			**LAmB**		
	**Proportion (%)**	**Cost (TL)**	Proportionate cost (TL)*	**Proportion (%)**	**Cost (TL)**	Proportionate cost (TL)*
**No baseline fungal infection**						
**Successful treatment**	24.58	11,551	2,839	29.62	19,492	5,774
**Mortality**	7.95	11,551	919	5.92	19,492	1,155
**Breakthrough fungal infection**	1.93	29,777	574	4.98	31,593	1,572
**Premature discontinuation**	9.88	30,179	2,982	6.64	29,784	1,976
**Persistent fever**	52.53	30,350	15,943	51.42	30,075	15,465
**Baseline fungal infection**						
**Successful treatment**	1.45	11,551	167	0.95	25,832	245
**Mortality**	0.00	0	0	0.00	0	0
**Persistent baseline fungal infection**	1.69	24,400	412	0.47	36,207	172
**Total**	100.00	--	23,835	100.00	--	26,358

*Individual costs may not sum to actual total as all figures are rounded to the nearest full Turkish Lira (TL).

The only univariate sensitivity analysis that resulted in a change in the study’s conclusion was in the alternative scenario created with the exclusion of fever resolution as part of the composite outcome measure. This resulted in voriconazole being the preferred alternative in all metrics considered (per patient treated TL13,960 vs. TL20,916; per successfully treated patient TL17,771 vs. TL25,511; per patient surviving TL13,971 vs. TL20,929, for voriconazole vs. LAmB). The change from a 5-point to a 4-point composite outcome measure also altered the overall success rate of each agent, from 26.02% to 78.55% for voriconazole and 30.57% to 81.99% for LAmB. All other one-way analyses did not change the study’s conclusion, but either slightly contracted or extended the economic preference for voriconazole. Removal of the cost of concomitant medications from the analysis also did not alter the study’s conclusion.

**Figure 3:**
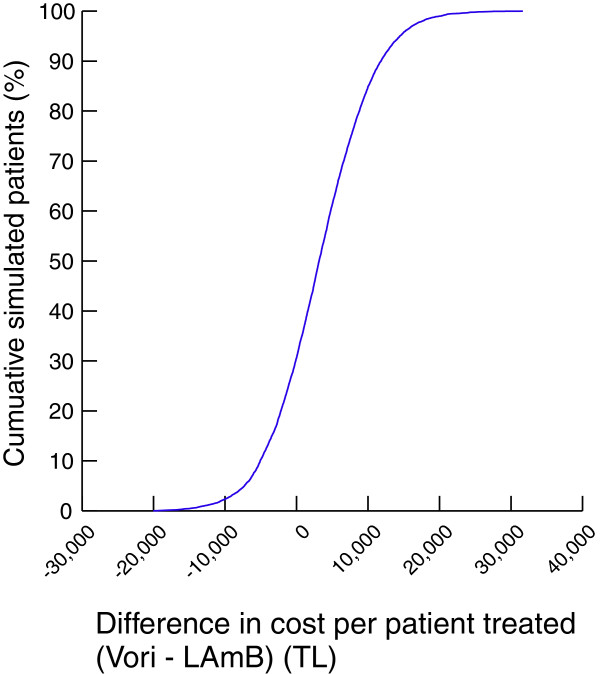
Probability of cost saving curve from the Monte Carlo Simulation sensitivity analyisis of voriconazole (Vori) versus liposomal amphotericin B (LAmB) per patient treated.

**Figure 4:**
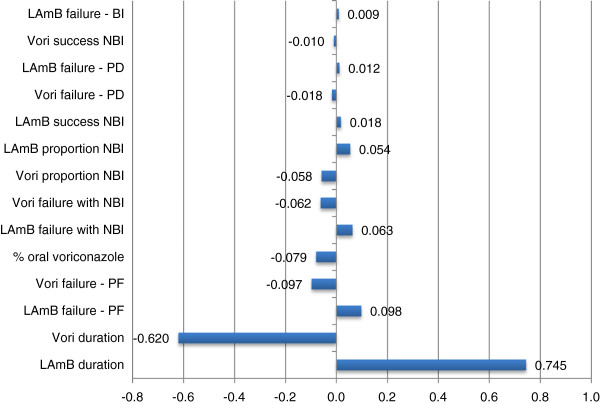
Tornado diagram of stepwise regression coefficients of effect of top 14 input variables on Monte Carlo Simulation (MCS) outcome (per patient treated).

Threshold analysis suggested the current model is highly sensitive to a change in treatment duration of either antifungal agent. An increase in voriconazole treatment duration by >1.2 days resulted in voriconazole no longer being the economically favourable option, from a per patient treated perspective. Likewise, a decrease in LAmB treatment duration by >1.0 days resulted in LAmB becoming the favourable alternative. The model was moderately sensitive to changes in drug acquisition costs. Voriconazole required an increase in list price of >32.4% or LAmB a decrease of >15.8% to change the study’s conclusion. Note that the change in list price of the antifungal agent was taken as an equivalent percentage change for both the vial and oral formulation of voriconazole (i.e. in determining the threshold of > 32.4%, both the vial and oral formulation were altered concurrently). Finally, MCS highlighted that when considering the two agents from a per patient treated perspective, there is a 69.4% probability of the analysis favouring voriconazole (Figures 3 and 4).

## Discussion

The current study suggests an economic advantage favouring the use of voriconazole over LAmB in the empiric treatment of febrile neutropenia in the Turkish or related healthcare settings. A major strength of this study was that it took into consideration the downstream consequences of treatment failures. Indeed, this is one of the few studies that have specifically targeted the choice of alternative antifungal agent based on the reason for treatment failure and/or organism cultured [10,18,19]. The use of an expert panel of clinicians from Turkey to determine the downstream effects of a treatment failure from the initial antifungal agent and, in validating the assumptions and clinical pathway as relevant and appropriate for the real-life setting within Turkey is another advantage.

While the assumption of mortality and other initial therapy failures occurring at the end of therapy may result in an overestimation of the total cost per patient treated, its overall impact on the study’s outcome is likely to be low given mortality rates were relatively similar in those receiving LAmB or voriconazole as initial therapy, and the assumption was applied equally across both agents with respect to initial therapy failure. The other assumption of requiring all patients with a baseline fungal infection that fail initial therapy to be considered to have had persistent fever should also have a minimal impact on the study’s outcome as the patient numbers in the baseline fungal infection branches, overall, was low.

The important differentiators of the Turkish healthcare system compared to other systems is highlighted through the results in this study; further emphasising that economic data from different countries with different healthcare systems and policies cannot be readily generalise to another country. Drug acquisition costs are relatively high whilst the daily cost per hospital bed is low in comparison to other western healthcare systems. A patient in the Turkish healthcare setting has their expenses subsidized by public insurance mechanisms, such as the SGK, which now covers the majority of the population [20]. Out of pocket healthcare expenses in Turkey have been reduced due to recent advances in the provision and financing of healthcare services (17.4% out of pocket in 2008) [20]. The current study will assist in formulary decision making in Turkey for the empirical use of voriconazole and LAmB and will also be an integral building block in decisions made within other regions with a similar healthcare system.

This study is different from earlier evaluations [11,21] in its use of a large multinational, multicentre randomized controlled trial [3] to populate the decision tree. Not only does this allow for comparative ease of generalization across similar healthcare systems, it allowed for theincorporation of the gold-standard 5-point composite outcome measure. In the current study, systemic review of the literature for evidence of effectiveness was not conducted. Whilst the randomized controlled study [3] used in the present economic analysis concluded that voriconazole is a suitable alternative to amphotericin B preparations, data from the US Food and Drug Administration (FDA) suggest that voriconazole was inferior to LAmB with respect to overall success rates (23.7% versus 30.1%, respectively) [22]. Had voriconazole been able to demonstrate non-inferiority to LAmB, the result from this evaluation would have economically favoured voriconazole. Hence the use of the data from this failed non-inferiority study is not expected to have an impact on the conclusion reached in the current economic evaluation.

Earlier economic analyses have utilized retrospective chart reviews with some also having limited scope in choice of both the definition of a successful outcome and in the adverse outcomes of interest [11,13,23]. An outcome that was investigated in several previous pharmacoeconomic studies was quality-adjusted life years (QALYs) [16,24,25]. This was not considered necessary or an appropriate endpoint in the current evaluation for two main reasons, namely the lack of a reasonable treatment duration to assess a significant QALY change (the non-inferiority trial used to populate the model did not include any long-term survival data beyond 7 days), and that the QALY gain/loss will be affected mainly by the patient’s underlying condition (e.g. acute myeloid leukaemia) and not by any effect from the choice of antifungal agent.

To assess the uncertainty involved in the determination of fever resolution as a component of the composite outcome, removing this scenario from the analysis was conducted. This alternate scenario resulted in voriconazole being the cost-effective alternative to LAmB for all three comparisons (per patient treated, survival and success). The significance of this finding is related to the difficulty in determining the resolution of fever for the purposes of classifying therapy as successful within a short time frame as seen in the pivotal efficacy studies of empiric antifungal therapy [17].

## Conclusion

The current study suggests that voriconazole would be the economically favourable alternative to LAmB when used as the initial agent in the empiric treatment of IFI. Our economic conclusion was moderately robust and is sensitive to treatment duration. The results from this evaluation will have implications for formulary decision making within Turkey and regions with a similar healthcare system, where treatment decisions in empiric antifungal therapy based on the full economic and clinical ramifications are not entirely clear.

## Abbreviations

IFI: Invasive fungal infection; LAmB: Liposomal amphotericin B; TL: Turkish Lira; ICU: Intensive care unit; G-CSF: Granulocyte colony-stimulating factor; FBE: Full blood examination; CT: Computed tomography; SGK: Sosyal Güvenlik Kurumu; BMT: Bone marrow transplant; MCS: Monte Carlo simulation; QALY: Quality-adjusted life year; BFI: Breakthrough fungal infection; PD: Premature discontinuation; PF: Persistent fever; Vori: Voriconazole; NBI: No baseline infection.

## Competing interests

This study was not funded by any pharmaceutical industry. DCMK has sat on advisory boards for Pfizer and received research/travel support (not related to the current work) from Pfizer, Roche, Merck, Novartis and Gilead Sciences. ECD has sat on advisory boards for Biocodex International and GlaxoSmithKline, received travel grants from Merck, GlaxoSmithKline, Pfizer and Novartis and is a speaker for GlaxoSmithKline. ES has received travel grants and honorarium as a speaker from Merck, Gilead Sciences, Pfizer and Novartis. AK has received travel grants and honorarium as a speaker from Merck, Gilead Sciences and Pfizer. SJT has no competing interests to declare.

## Authors’ contributions

SJT was involved in the concept development, protocol design, analysis and manuscript preparation. ES was involved in the concept development, protocol design and manuscript review. AK was involved in the concept development, protocol design and manuscript review. DA was involved in the concept development, protocol design, analysis and manuscript review. ECD was involved in the concept development, protocol design and manuscript review. DCMK was involved in the concept development, protocol design, analysis and manuscript preparation. All authors read and approved the final manuscript.

## Authors’ information

Ener C. Dinleyici and David CM Kong are co senior authors.

## Pre-publication history

The pre-publication history for this paper can be accessed here:

http://www.biomedcentral.com/1471-2334/13/560/prepub
